# First report of the stubby-root nematode *Nanidorus minor* infecting *Paspalum vaginatum*, seashore paspalum grass in Georgia, USA

**DOI:** 10.21307/jofnem-2020-106

**Published:** 2020-11-10

**Authors:** Ganpati B. Jagdale, Fereidoun Forghani, Katherine Martin, Abolfazl Hajihassani, Alfredo Dick Martinez-Espinoza

**Affiliations:** 1 Extension Nematology Lab, Department of Plant Pathology, University of Georgia, Athens, Georgia, 30602; 2 Department of Plant Pathology, University of Georgia, Tifton, GA, 31794; 3 Department of Plant Pathology, University of Georgia, Griffin, GA, 30223

**Keywords:** Diagnosis, Georgia, *Nanidorus minor*, Paspalum grass, Turfgrass

## Abstract

We found that *Nanidorus* spp. was pathogenic to seashore paspalum (*Paspalum vaginatum*) turfgrass as its population increased from 100 to 2,080 nematodes per pot 180 days after inoculation under greenhouse conditions. Morphological measurements of adult females were similar to those described for *N. minor*. Molecular analysis also confirmed the morphological identification by targeting three different regions of the genomic DNA. Three primer pairs targeting 18S rDNA (360F/932R), 28S rDNA (D2A/D3B) and ITS1 rDNA (BL18/5818) were used in singleplex PCR. Forward and reverse sequences of each individual primer set were then subjected to multiple alignment and the complimentary sequences were assembled into a consensus sequence. Upon nucleotide blast on the NCBI website, they were all confirmed to be *N. minor*. A one-step multiplex PCR method using specific primers and a fragment size of 190 bp also confirmed the identity of *N. minor*. To the best of our knowledge, this is the first report of *N. minor* infecting seashore paspalum turfgrass in Georgia.

Stubby-root nematodes, *Nanidorus* spp., are one of the most common plant-parasitic nematodes found associated with different species of turfgrasses in Georgia (Unpublished data, Nematode Diagnostic Laboratory, University of Georgia Database, 2000 through 2019). In March 2019, five soil samples were submitted to the Nematode Diagnostic Laboratory, University of Georgia, Athens for diagnosis of plant-parasitic nematodes associated with seashore paspalum (*Paspalum vaginatum* Swartz) grown on a turf farm located in South Georgia. While processing these samples, 112 stubby-root nematodes per 100 cm^3^ of soil were found to be associated with this turfgrass, which is currently grown in many sport fields and golf courses in Georgia and Florida. This prompted us to first study the pathogenicity of *Nanidorus* spp. to seashore paspalum turfgrass and second to determine the species identity using both molecular and morphological techniques. Accurate species identification of the nematodes can be helpful to develop effective control strategies. For the pathogenicity study, nematode-free seashore paspalum plants were grown from sprigs in five clay pots containing 500 g sandy-loam soil and allowed to establish for two weeks. After two weeks, three pots were inoculated with 100 handpicked, mixed-stages of juveniles and females of *Nanidorus* spp. per pot extracted from original soil samples using the decanting and flotation technique (Jenkins, 1964). The remaining two pots were maintained as non-inoculated controls. The population density of the nematode was determined 180 days after inoculation under greenhouse conditions at 25  ± 1°C. Results showed that *Nanidorus* spp. could infect seashore paspalum as the average nematode population increased from 100 to 2,080 nematodes per pot. The roots of infected grass plants exhibited ‘stubby’ appearance and stunted growth compared to the controls that had healthy root systems (Fig. 1). Morphological and molecular analyses were conducted in the Nematology Laboratory, University of Georgia, Tifton, GA to identify nematode species associated with seashore paspalum turfgrass. Because nematode males were not found in the samples, female specimens were used for identification. Measurements (mean, range) of adult females (*n* = 16) included body length 664.5 (562.1-768.9) μm; body width 31.9 (27.2-36.4) μm; onchiostyle 31.6 (31.0-33.4) μm; anterior end to esophagus-intestinal valve 115.0 (99.6-126.1) μm; vagina length 8.4 (7.2-10.3) μm; *a* 21.1 (15.9-27.3) μm; *b* 5.0 (4.6-6.0) μm; and *V* 51.4% (47.5-54.1%) μm. Measurements of morphological characters of adult females were similar to *N. minor* (Colbran) Siddiqi (Decraemer, 1995; Hajihassani et al., 2018a). Due to similar morphometrics of *N. minor* and closely related species of *Nanidorus*, a molecular analysis was first performed to confirm the morphological identification by targeting three different regions of the genomic DNA. Briefly, DNA samples were extracted from single stubby-root nematodes (*n* = 5) using Extract-N-Amp™ Tissue PCR Kit (Sigma-Aldrich Co. LLC, St. Louis, MO‎; USA) following kit protocol with modifications. Individual nematodes were transferred into a sterile petri dish in 5 µL water and cut into 2 to 3 pieces using pipette tip under an inverted microscope. Subsequently, each individual nematode mix was transferred into a sterile 1.5 mL microcentrifuge tube containing 20 μL of extraction buffer with 5 µL of Tissue Preparation Solution from the kit. After careful vortexing, the mixture was incubated at room temperature (23 ± 2°C) for 10 min and then at 95°C for 3 min. Finally, 20 µL of neutralization solution was added to the tube and vortexed briefly. This DNA extract was stored at −20°C and used as DNA template for PCR reactions in 2 µL aliquots. Three primer pairs targeting 18S rDNA (360F/932R), 28S rDNA (D2A/D3B) and ITS1 rDNA (BL18/5818) (Riga et al., 2007; Duarte et al., 2010; Ye et al., 2015) were used in singleplex PCR (Hajihassani et al., 2018a). The conditions used in PCR reactions were an initial denaturation at 94°C for 2 min followed by 40 cycles of (94°C for 45 sec, 53°C for 30 sec, and 72°C for 2 min) and a final 10 min extension at 72°C. The 557-, 750- and 860-bp PCR products of 360F/932R, D2A/D3B, and BL18/5818, respectively, were then subjected to purification and Sanger sequencing (Genewiz Inc., South Plainfield, NJ, USA). Forward and reverse sequences of each individual primer set were then subjected to multiple alignment and the complimentary sequences were assembled into a consensus sequence. Upon nucleotide blast on the National Center for Biotechnology Information (ncbi.nlm.nih.gov) website, they were all confirmed to be *N. minor*. The top pairwise identity with *N. minor* references was 100% for 360F/932R (accession no. MT36688) and BL18/5818 (MT372094), and 99.73% for D2A/D3B (MT371258). In contrast, the highest identity to other members of the *Nanidorus* sp. was in the range of 96% or lower. A one-step multiplex PCR method was also performed using specific primers as described by Huang et al. (2019). A fragment size of 190 bp was observed only for *N. minor*, confirming the identity of this species. To the best of our knowledge, this is the first report of *N. minor* infecting seashore paspalum turfgrass in Georgia. This nematode species was reported to cause damage on St. Augustine grass, switchgrass, Vidalia onion and field and sweet corn in Georgia (Hajihassani et al., 2018b).

**Figure 1:**
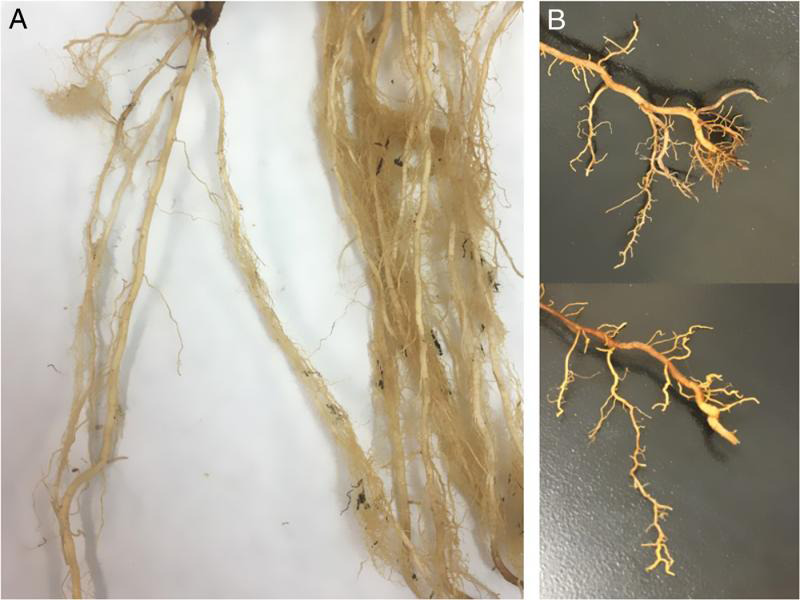
Healthy roots of Seashore Paspalum grass collected from pots not-inoculated with the stubby-root nematode, *Nanidorus minor* (A), and roots collected from *N. minor*-inoculated pots showing ‘stubby’ appearance or stunted growth (B).
